# Cesarean Delivery in a Patient With Segawa Syndrome (Dopamine-Responsive Dystonia) Under Epidural Anesthesia: A Case Report

**DOI:** 10.7759/cureus.107520

**Published:** 2026-04-22

**Authors:** Eunkyung Choi, Hyeonseung Yi, Juhee Min, Deokhee Lee

**Affiliations:** 1 Department of Anesthesiology and Pain Medicine, Yeungnam University College of Medicine, Daegu, KOR

**Keywords:** anesthesia, cesarean section, dystonia, epidural, levodopa, pregnancy

## Abstract

We report a case of a pregnant patient with dopamine-responsive dystonia (DRD; Segawa syndrome) who underwent cesarean delivery under epidural anesthesia. The patient continued levodopa therapy throughout pregnancy and the perioperative period without interruption. Anesthetic management was carefully tailored to prevent exacerbation of dystonic symptoms. Hemodynamic stability was maintained throughout surgery, and no perioperative neurological complications were observed. This case highlights the feasibility and safety of neuraxial anesthesia with continued dopaminergic therapy and underscores important anesthetic considerations for cesarean delivery in patients with DRD.

## Introduction

Dopamine-responsive dystonia (DRD), also known as Segawa syndrome, is a rare genetic movement disorder with an estimated prevalence of 0.5-1 case per million individuals [1,2]. It usually presents in childhood or adolescence with progressive dystonia and gait abnormalities exhibiting diurnal variation [1]. DRD is characterized by a dramatic response to low-dose levodopa therapy, reflecting impaired dopamine biosynthesis with preserved nigrostriatal neuronal integrity [3]. Females are reported to be affected more frequently than males [2]. Only a limited number of reports have described cesarean delivery in patients with DRD [4,5]. Accordingly, this report presents cesarean delivery under epidural anesthesia in a patient with Segawa syndrome, with a particular focus on continuous levodopa therapy and perioperative hemodynamic management as distinguishing clinical features, and reviews relevant anesthetic considerations.

## Case presentation

A 31-year-old primigravida woman (height 161 cm, weight 66 kg) with GTP cyclohydrolase 1 (GCH1)-related DRD was scheduled for elective cesarean delivery at 36 + two weeks of gestation based on maternal request in the absence of obstetric indications for surgical delivery. She had a 20-year history of progressive lower-limb rigidity and dystonic posturing that was unresponsive to rehabilitation therapy. Her symptoms had been well controlled with levodopa 62.5 mg twice daily since the age of 23 years. She was started on levodopa therapy at the age of 23 years based on clinical suspicion of DRD, and the diagnosis was subsequently confirmed at the age of 28 years following detection of a heterozygous mutation in the GCH1 gene.

**Figure 1:**
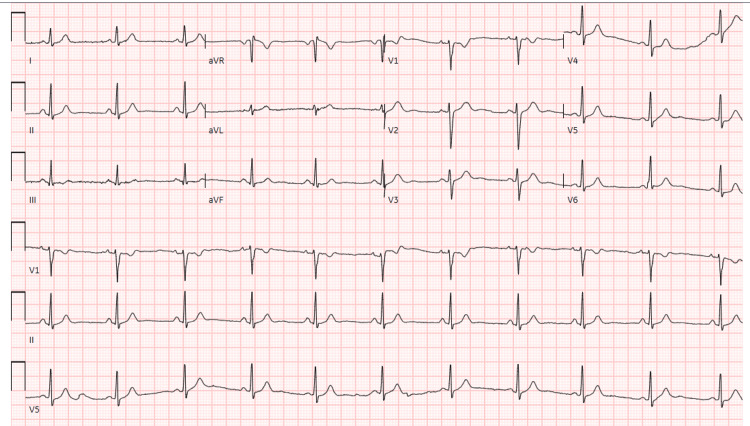
Preoperative electrocardiography showing normal sinus rhythm

**Figure 2:**
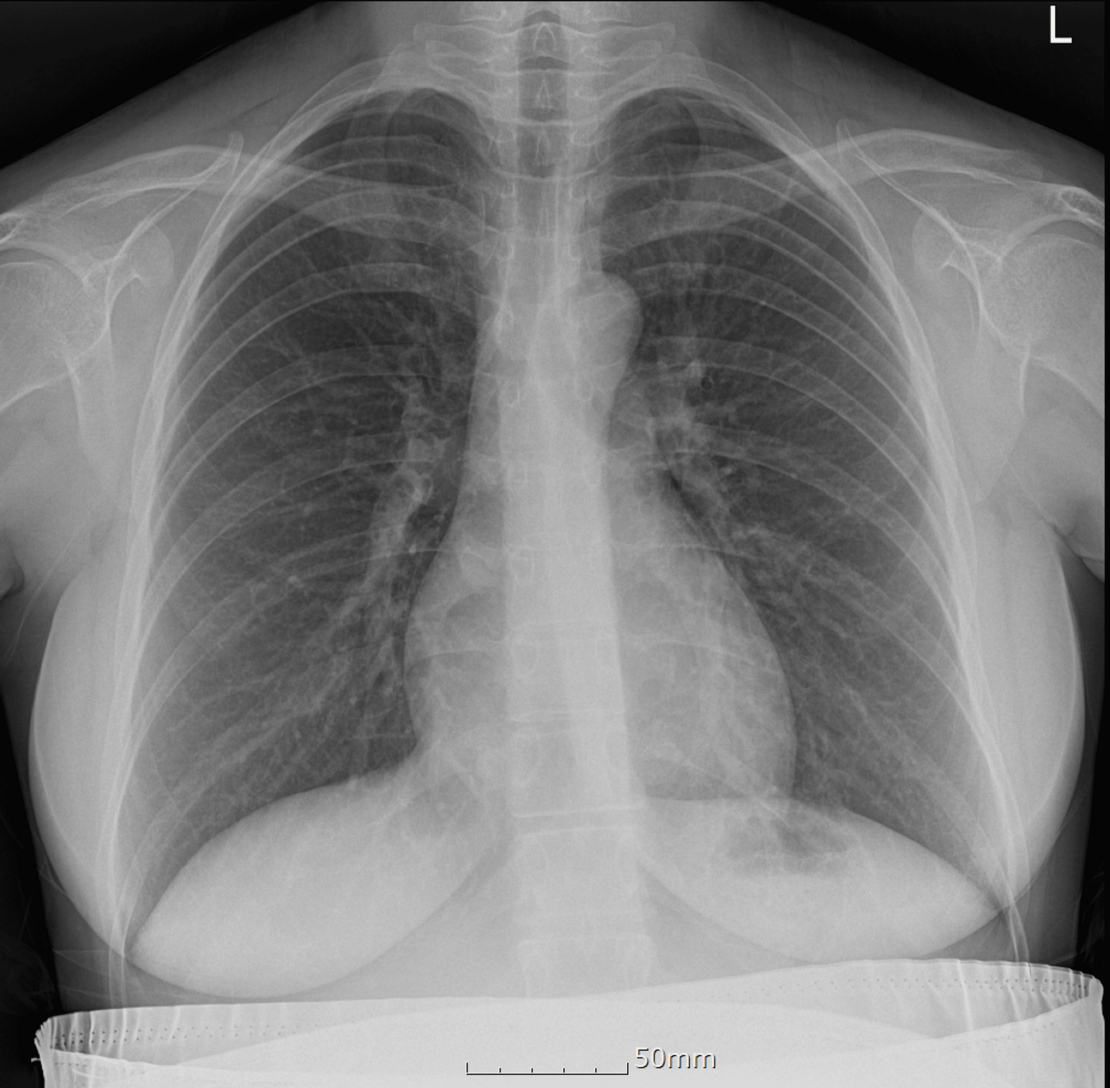
Preoperative chest radiography showing no abnormal findings

Apart from a transient fetal cavum vergae noted at 24 weeks of gestation, the pregnancy was uneventful, and her neurologic symptoms did not worsen. Preoperative evaluation revealed normal cardiopulmonary function, a Mallampati grade I airway, and an American Society of Anesthesiologists physical status II. Laboratory findings were within normal limits (Table 1). Preoperative electrocardiography (Figure 1) and chest radiography (Figure 2) were unremarkable.

**Table 1:** Routine laboratory tests within normal range ALT, alanine aminotransferase; AST, aspartate aminotransferase; BUN, blood urea nitrogen; T4, thyroxine; TSH, thyroid-stimulating hormone

Parameter	Result	Reference range	Unit
White blood cells	5.83	4-10	× 10³/µL
Hemoglobin	13.3	12-16	g/dL
Platelet	225	140-440	× 10³/µL
Sodium	136	135-145	mEq/L
Potassium	4.5	3.5-5.5	mEq/L
Chloride	105	98-110	mEq/L
Glucose	89	74-99	mg/dL
AST	24	10-35	IU/L
ALT	10	0-40	IU/L
BUN	9.5	8-23	mg/dL
Creatinine	0.52	0.5-0.9	mg/dL
TSH	1.25	0.27-4.2	µIU/mL
Free T4	0.99	0.92-1.68	ng/dL

On the day of surgery, the patient took her regular morning dose of levodopa (62.5 mg). Upon arrival in the operating room, baseline vital signs were stable, with a blood pressure of 102/59 mmHg, a heart rate of 64 beats per minute, and an oxygen saturation of 98%. An epidural block was established at the L3-4 level using an 18-gauge Tuohy needle, with the patient in the left lateral decubitus position. After infiltration with 2% lidocaine, a test dose was administered. Correct epidural placement was confirmed by the absence of hemodynamic or clinical changes. Subsequently, 20 mL of 2% lidocaine with epinephrine was injected, targeting a T4 sensory level for cesarean delivery. Oxygen was administered via face mask at 5 L/min, and 500 mL of balanced crystalloid solution was infused before surgery as a preload prior to epidural anesthesia.

During surgery, maternal hemodynamics remained stable (SBP 100-120 mmHg, DBP 50-60 mmHg, HR 60-80 bpm, SpO₂ 100%). A male infant weighing 2,420 g was delivered 10 minutes after skin incision, with Apgar scores of 8 and 9 at one and five minutes, respectively. Oxytocin (60 U in 100 mL normal saline) and carbetocin (100 µg IV) were administered to promote uterine contraction. A transient decrease in blood pressure to 95/51 mmHg was treated with IV phenylephrine 50 µg, resulting in rapid recovery to 110/60 mmHg within a few minutes. Estimated blood loss was 600 mL. Intraoperative fluids included 300 mL Volulyte and an additional 600 mL plasma solution, with a total urine output of 70 mL. The duration of surgery was 87 minutes.

In the post-anesthesia care unit, the sensory block had regressed to the T6 level on arrival. A half-tablet of levodopa/benserazide 100/25 mg (50 mg levodopa) was prepared for potential rescue treatment to allow rapid control of acute dystonic symptoms, but was not required, as no dystonia occurred. The sensory block remained at the T6 level for 110 minutes. The patient remained hemodynamically stable, with a post-anesthesia recovery score ≥8.

Postoperative analgesia was provided using epidural patient-controlled analgesia with ropivacaine 0.75% (60 mL) and fentanyl 50 µg in 240 mL normal saline (total volume 300 mL). The infusion was delivered at a basal rate of 5 mL/hr, with a bolus dose of 2 mL and a lockout interval of 20 minutes. Pain was well controlled; however, the epidural infusion was discontinued after two days because of right-leg numbness. A postoperative neurological assessment revealed that the symptoms were consistent with a dermatomal sensory block related to epidural analgesia, without evidence of dystonia or neurological complications, and the symptoms resolved after discontinuation of the epidural infusion. Postoperative pain was subsequently managed with systemic analgesics, with adequate pain control. The patient and neonate were discharged on postoperative day 5 without complications. At one-week follow-up, the patient continued levodopa twice daily with no recurrence of dystonic symptoms.

## Discussion

This case describes a patient with DRD who successfully underwent cesarean section under epidural anesthesia. The focus of this report is the use of epidural anesthesia while maintaining continuous levodopa therapy throughout the perioperative period. This approach allowed stable hemodynamic control, avoidance of potential drug interactions associated with general anesthesia, and real-time neurological assessment during surgery.

DRD, also known as Segawa syndrome, is a rare movement disorder characterized by dystonic motor manifestations, parkinsonian features, and a pronounced therapeutic response to low-dose levodopa, with diagnosis primarily based on these characteristic manifestations [1]. Dystonia and mild parkinsonism typically exhibit diurnal variability, marked by evening deterioration and restoration of function after sleep [3]. Approximately 80% of cases follow an autosomal dominant inheritance pattern and are associated with pathogenic variants in the GCH1 gene, a key enzyme in dopamine biosynthesis, resulting in reduced dopamine production within the basal ganglia [6,7]. Autosomal recessive forms, caused by mutations in the tyrosine hydroxylase gene, are rare and are generally associated with a more severe phenotype due to impaired catecholamine synthesis [8,9].

DRD shares certain features with Parkinson’s disease, particularly dopamine deficiency and the therapeutic effectiveness of levodopa in restoring dopaminergic function [10]. However, unlike Parkinson’s disease, which involves progressive dopaminergic neuronal loss, DRD results from a genetically determined impairment in dopamine synthesis with preservation of nigrostriatal pathways. Owing to this distinct pathophysiological mechanism, DRD typically manifests during childhood, shows prominent diurnal fluctuation, and demonstrates a dramatic response to levodopa, in contrast to the gradual progression and variable treatment responsiveness observed in Parkinson’s disease [10]. In this case, the patient developed progressive lower-limb dystonia at approximately 10 years of age, and her symptoms have been well controlled since initiation of low-dose levodopa therapy (62.5 mg twice daily) in her early 20s.

Several anesthetic considerations should be addressed in patients receiving chronic levodopa therapy for DRD. Levodopa is converted to dopamine by decarboxylase enzymes in the central nervous system, systemic circulation, and other tissues [11]. Increased endogenous dopamine may augment cardiac output via inotropic and chronotropic mechanisms, with a possible risk of provoking myocardial irritability. In addition, chronic dopaminergic stimulation may suppress norepinephrine production through negative-feedback inhibition, leading to relative hypovolemia and an increased risk of orthostatic hypotension. Accordingly, careful intravascular volume management and close hemodynamic monitoring are essential during anesthesia. In addition, dopamine-antagonistic agents such as phenothiazines and metoclopramide warrant avoidance or cautious use due to the risk of exacerbating dystonia and precipitating extrapyramidal symptoms [4].

In cesarean delivery, volume loading is commonly performed before or immediately after anesthetic induction to reduce neuraxial anesthesia-associated hypotension and maintain adequate uteroplacental perfusion [12]. In our patient, approximately 10 mL/kg of IV fluid was administered at the initiation of epidural anesthesia as a preventive measure. Despite this, maternal hypotension occurred following sympathetic blockade and was effectively treated with IV phenylephrine. Phenylephrine is recommended as the first-line vasopressor during cesarean delivery because it provides better fetal acid-base status than ephedrine [13,14]. As a direct α1-adrenergic agonist, phenylephrine may provide more predictable hemodynamic control than indirect sympathomimetic agents in patients receiving chronic levodopa therapy.

Considering anesthetic implications related to dopaminergic dysfunction and chronic levodopa therapy, both general and regional anesthesia have been reported to be safely performed in patients with DRD when appropriate precautions are taken [5,15]. In this patient, epidural anesthesia was selected to avoid potential drug interactions associated with general anesthesia and to allow continuous neurological assessment during surgery. General anesthesia with adjuncts such as nitrous oxide was considered but not selected, given these concerns. In addition, epidural anesthesia allows gradual titration of the sensory block, thereby reducing the risk of abrupt hemodynamic changes compared with spinal anesthesia [16], which may be particularly relevant in patients receiving chronic levodopa therapy with potentially unpredictable hemodynamic responses. This may help mitigate sudden sympathetic blockade and associated hypotension. Regional anesthesia is also advantageous in minimizing exposure to dopamine-antagonistic agents, reducing central nervous system interactions, and providing effective postoperative analgesia with a lower risk of residual neuromuscular effects. However, neuraxial techniques may be associated with sympathetic blockade and hypotension, requiring careful hemodynamic monitoring and adequate volume loading [15]. In addition, underlying movement disorders such as dystonia may complicate patient positioning during neuraxial anesthesia and should be carefully considered during anesthetic management. No positioning-related complications were observed, likely due to adequate control of dystonic symptoms; however, careful positioning remains important in such patients. Although no definitive evidence favors one anesthetic technique over another, anesthetic management should be tailored to the patient’s neurological status and surgical requirements to maintain hemodynamic stability and uninterrupted dopaminergic therapy. Epidural anesthesia may not be suitable in patients with poorly controlled dystonia or significant hemodynamic instability.

The available literature concerning pregnancy in individuals with DRD remains limited, largely comprising single case reports. During pregnancy, dopaminergic requirements may remain stable or increase because of physiological and hormonal changes. Continuation of levodopa therapy is generally recommended, as discontinuation may result in worsening dystonia, functional impairment, or akinetic crisis. In this context, the decision to continue levodopa therapy was based on a careful assessment of the balance between maternal benefits and potential fetal risks. Serum drug levels were not measured; instead, therapy was guided by clinical neurological assessment and symptom stability. The patient remained neurologically stable, and no adverse maternal or fetal outcomes were observed. In the present case, levodopa therapy was maintained throughout pregnancy without dose escalation, and no obstetric or neonatal complications attributable to the medication were observed. Existing reports suggest that levodopa can be continued safely during pregnancy and delivery, with no consistent evidence of adverse fetal outcomes attributable to the medication [4,17,18]. However, Sinha and Hartsilver reported a case in which pregnancy and delivery were managed without levodopa because of concerns regarding fetal safety [5]. According to the British National Formulary, levodopa monotherapy or combination therapy with peripheral dopa decarboxylase inhibitors is contraindicated during pregnancy [19]. Therefore, decisions regarding levodopa use should be individualized, balancing maternal disease control against potential fetal risk.

Neonatal follow-up beyond one week was limited in this case. Chromosomal analysis at one month revealed no GCH1 gene mutation, and no abnormalities were observed during the early neonatal period. However, information regarding breastfeeding and levodopa exposure during lactation was not available.

## Conclusions

This case demonstrates that epidural anesthesia for cesarean section can be safely administered in patients with DRD/Segawa syndrome who continue levodopa therapy throughout pregnancy. Careful perioperative planning, including maintenance of dopaminergic treatment, avoidance of dopamine-antagonistic agents, and close hemodynamic monitoring, is essential to ensure maternal and fetal safety. Selection of an appropriate anesthetic technique, particularly epidural anesthesia when feasible, is also important. Further accumulation of clinical data is required to establish evidence-based guidelines for anesthetic and obstetric management in this rare population.
